# 5. RETHINKING THE TAXONOMY, COURSE, AND OUTCOME OF PSYCHOSES: DIMENSIONAL, LATENT TRAJECTORY, AND TRANSDIAGNOSTIC APPROACHES

**DOI:** 10.1093/schbul/sby014.012

**Published:** 2018-04-01

**Authors:** William T Carpenter

**Affiliations:** University of Maryland School of Medicine

## Abstract

**Overall Abstract:** Evidence continues to accumulate on heterogeneity in phenomenology, course and outcome of non-affective and affective psychotic disorders. Both DSM and ICD classification systems have evolved to include a large number of categories of psychosis. However, doubt remains about this categorical approach because of high comorbidity, common etiological factors and the absence of zones of relative rarity between categorical diagnoses. Some authors have nevertheless argued that categorical representations of psychosis may still be of clinical utility if used in combination with dimensional indicators.

It is now widely accepted that psychotic symptoms partition into several symptom dimensions that would support the heterogeneity of psychotic disorders. However, there is no consensus on the exact number of these dimensions, with previous factor-analytic work pointing towards models with two to twelve specific symptom dimensions. However, recently, there has been evidence for a transdiagnostic dimension underlying affective and non-affective psychotic symptoms in schizophrenia, schizoaffective and bipolar disorder that challenges their classification as distinct diagnostic constructs. There is also considerable heterogeneity in clinical course and outcome of psychotic disorders, but how to best map and model this over time remains to be established. Taken together, this presents significant challenges for the classification of psychotic disorders as separate diagnostic entities.

This symposium brings together international researchers at the forefront of research into the phenomenology, course and outcome of psychotic disorders. Roman Kotov will present novel data on symptom dimensions and examines the course of these dimensions in an epidemiologic cohort of 628 first-admission inpatients with psychosis interviewed 6 times over two decades in the Suffolk County Mental Health Project. Craig Morgan will report new findings from the 10-year follow-up of the Aetiology and Ethnicity in Schizophrenia and Other Psychoses (AESOP-10) study, an epidemiological cohort of 552 patients with a first episode psychosis, using a data driven approach to identify latent trajectory classes to account for heterogeneity in patterns of change in psychotic symptoms over time and characterize these trajectories with the WHO classification, baseline demographic characteristics and diagnoses. Ulrich Reininghaus will present novel data from the Bipolar-Schizophrenia Network on Intermediate Phenotypes (B-SNIP) consortium to investigate whether there is a transdiagnostic dimension cutting across symptoms of schizophrenia, schizoaffective disorder and psychotic bipolar I disorder. Diego Quattrone will report recent findings from EU-GEI Functional Enviromics Study on genetic and socio-environmental factors associated with transdiagnostic and specific symptom dimensions of non-affective and affective psychosis. Robin Murray will discuss these findings in the context of new challenges in the field and directions for future research.

